# Challenges of Measuring a Faculty Member Activity in Medical Schools

**Published:** 2011-03-01

**Authors:** A Mohammadi, R Mojtahedzadeh, S H Emami Razavi

**Affiliations:** 1 Educatinal Development Centre, Tehran University of Medical Sciences, Tehran, Iran; 2 Faculty of Medicine, Tehran University of Medical Sciences, Tehran, Iran

**Keywords:** Evaluation, Faculty members, Activity, Metrics system

## Abstract

**Background:**

One of the features of Mission Based Management is measuring the activities of faculty members and departments and their contributions to the school's mission. As it is important to assess the school's readiness for such a system, in this study we assessed the view points of Tehran Medical School's department chairs about faculty members’ activities.

**Methods:**

We used focus group technique to identify participants' view points. We divided 30 department chairs into homogenous groups of 4-6. After a brief introduction, the moderator presented questions to determine the participants' idea and a secretary recorded them. We categorized view points into main themes and subthemes.

**Results:**

Ninety three percent of chairs participated in the sessions. Department chairs' view points were categorized into 3 main themes: "system is effective and important", "system is effective and important but some challenges should be considered" and "system is ineffective and should not be implemented". Subthemes included chairs' concerns, views, fears and reasons.

**Conclusions:**

The results of the study provided reliable information about department chairs' concerns and reactions to this system. Finally, we determined points of strengths and anticipated threats for developing a faculty member activity measurement system.

## Introduction

In 1998, Mission Based Management (MBM) was introduced as a solution to develop mission-driven decision making, ensure organizational accountability and distribute resources in alignment with school's missions.[1][2] MBM has some core features, among which measuring faculty members and departments' activities and their contributions to school's missions, is considered to be a basic component.[1][2] Reports contain guidance in developing metric systems for education,[3] research [4] and patient care.[5] Developing such metric systems in medical schools, by itself, are considered to be of great benefit for mission enhancement.[6][7] Some of these benefits are: tracking school and departments' performance overtime,[5] providing clear measurable expectations and standards,[1][2][3][5] offering incentives for excellence, measuring and valuing education/teaching activities,[3][4] career counselling of faculty members about allocation of their time to different missions,[3] providing a management tool for evaluating departments’ and individual faculty members’ performances and their comparison [4][7] and so on. To develop a metric system in a medical school, it is recommended to perform a participative process and provide relevant opinion leaders with the chance of expressing their concerns.[1][5][6]

In Medical School of Tehran University of Medical Sciences, there was no system for measuring faculty members and departments' activities. There was no data about concerns and view points to be considered in designing and implementing such a system. So we performed this study to assess view points of department chairs on this issue.

## Materials and Methods

We used focus group technique to identify view points of department chairs of Medical School of Tehran University of Medical Sciences about measuring faculty members' activities. This study was performed between May to July 2008.

We divided school's 30 educational departments into homogenous groups of 4-6 considering their specialty (e.g. basic sciences/clinical or surgical/nonsurgical).

We held 1 to 2 sessions for each group. Participants were invited by school's dean who participated in all sessions. The same moderator managed all sessions.

In each session we spent 30 minutes to present a brief introduction to the issue and clarify the importance and aims of the session. Then the moderator presented a predefined set of questions to determine participants' ideas regarding metric system of faculty members' activities. He guided and encouraged the group to present their ideas, ask questions, explain their reasons and comment on others points of view.

A secretary recorded the view points and also typed them down in a Microsoft Office Word sheet projected on board to be in full view for the group.

For analysis, we reviewed department chairs' view points thoroughly and categorized them into main themes. The reasons for each theme group were classified into subthemes too.

## Results

Eleven sessions were held and each one lasted for about 2-2.5 hours. Ninety three percent of chairs (28 out of 30 departments) participated in sessions. Department chairs' view points were categorized into 3 main themes: i) Faculty members’ activities measurement is effective and important (69% of participants); ii) Faculty members’ activities measurement is effective and important but some challenges should be considered (20.7% of participants) and iii) Faculty members’ activities measurement is ineffective and should not be implemented (10.3% of participants). Table 1, Table 2, and Table 3 show each theme-category's subthemes respectively.

**Table 1:** Subthemes related to the theme-category 1: "Faculty members’ activities measurement is effective and important". Tehran Medical School's department chairs' view points, reasons and comments for effectiveness and importance of the faculty members' measurement system.

**Subthemes relevant to theme category 1: "Faculty members' activities measurement is effective and important" **
The system is effective because of:
Increasing chairs' authority
Tracking faculty members' activities to decide on:
Contribution of faculty members and departments to different missions
Budget allocation and salary payment
Faculty members' tenure and promotion
Rewarding system
Determining faculty members track
Increasing faculty members' responsibility
Providing the possibility of measuring effective physical presence of faculty members
Providing the possibility of measuring all activities, including very routine ones
Rewarding and emphasizing on educational/teaching activities
Clarifying expectations
Enhancing traditional management tools

Also department chairs had some suggestions as follows: i) Emphasizing on positive incentives and rewards rather than negative ones, ii) Encouraging full time faculty members, iii) Establishing an atmosphere of trust by self reporting of activities, iv) Validating faculty members' self-reported activities by ward/department chairs, v) Valuing educational/teaching activities, vi) Implementing the system gradually and vii) Developing a web-based system.

**Table 2:** Subthemes related to the theme-category 2: "Faculty members’ activities measurement is effective and important but some challenges should be considered". Tehran Medical School's department chairs' expressed challenges to be considered for developing faculty members' measurement system.

** Subthemes relevant to theme category 2: "Faculty members’ activities measurement is effective and important but some challenges should be considered"**
Challenges to be considered for developing the system:
Necessity to revise department chairs' selection process beforehand
Necessity to provide more authority for department chairs beforehand
Necessity to consider quality of activities along side with their quantity
Necessity to pay attention to negative effects of the system on faculty members' income, tenure and promotion
Possibility of encountering conflicts between metric system and other existing performance measuring systems
Possibility of defining high expectations by school that results in faculty members' overload of work
Possibility of faculty members' score seeking
Possibility of faculty members' gaming
Cultural change challenges
Low faculty members' incentives
Necessity to provide required facilities in the departments beforehand

**Table 3:** Subthemes related to the theme-category 3: "Faculty members’ activities measurement is ineffective and should not be implemented". Tehran Medical School's department chairs' view points and reasons for ineffectiveness of the faculty members' measurement system.

** Subthemes relevant to theme category 2: "Faculty members’ activities measurement is ineffective and should not be implemented"**
The system is ineffective because of:
Differences among disciplines, specially regarding income (e.g. basic sciences versus surgeons)
Faculty members' responsibilities not legally clear
Not having an environment of trust in the school and within departments
No difference between highly active faculty members and their colleagues
Not a unique template works for all departments
Believing in pure qualitative evaluation instead of quantitative approach
Not having a good experience about previous evaluation systems
Not believing in evaluation systems at all.

## Discussion

Several reports showed that schools would encounter challenges for developing metric systems.[6][7][8][9][10][11][12] Some of the challenges and view points emerged from our study are also discussed in literature. In today's modern medical schools, educational activities need to be re-enhanced more.[8][9] In our study, department chairs emphasized on valuing educational/teaching services too.

Our chairs had worrying concepts about a pure quantitative metric system replacing existing qualitative ones and asked for integrating school's existing qualitative evaluation systems with activities' metric system, as it appears in the literature too.[3][6][7][9] In one study, some faculty members expressed their fear about looking underproductive.[7] Participants in our study expressed the same fear regarding their income and promotion. This is an important issue to be considered while developing the system.

One significant challenge is encountering a persistent general resistance by faculty members and chairs to the system.[7] We had the same expressed resistance, especially by basic sciences departments' chairs. We should clarify school's plan for developing system to construct an atmosphere of trust through a participative process.

Other points to be considered are faculty members' gaming in their activities' self-reports and their score seeking (by performing high-weighted activities and thus forgetting low-weighted but essential ones).[10] Our chairs had the same fears. So self-reported activities should be reviewed and verified by department chairs.[7][11][12]

Some of our chairs had the concern that metric systems would be in conflict with other performance measuring reports such as assessing time sheet, and tenure and promotion processes. The same concern is reported in another experience. Providing the opportunity of assessing the metric system's reports to be congruent among other school's reports would be helpful.[12]

On the other hand, our department chairs emphasized that system implementation and linking its reports to critical decision making would happen slowly and deliberately. We believe that this approach would greatly help school's dean encounter many of above mentioned problems. Lessons learnt from other experiences provide related evidence.[6][7]

Some of sub-themes emerged from our study were not reported in literature. Maybe these were considered to be so specific for our school. For example, Tehran Medical School's department chairs were selected through an election process within departments. Some chairs had severe criticism to election process. Among expressed expectations, there were some exaggerated and general ones that should be modified. For example, "enhancing traditional management tools" was a general phrase, addressing some managerial challenges within the school, which would not be met in a metric system. Mallon and Jones (2002) addressed these expectations and declared that the metrics process cannot be a onetime fix or cure-all.[6]

The results of the study would be helpful in devising a comprehensive faculty members' activities metric system. School's dean had no reliable information of how department chairs would react to such a system and what would be their concerns and fears. Also chairs were satisfied that school's dean had provided them the chance of expressing their viewpoints and participating in the metric system developing process.

Also we determined points of strengths and threats for developing a faculty member’ activity measurement system, based on department chair' views.

Points of strengths were that most of the chairs believed in need and effectiveness of such a system, they had a positive approach, they thought that an objective and trustful system could have a great positive impact and school's dean tended to support the system.

Points of threats were that some chairs were pessimistic about new evaluation systems, some did not believe in evaluation at all, they were worried about their department and their own results, some had overestimated expectations and some departments lacked enough personnel and equipment.

## References

[1] Ridley GT, Skochelak SE, Farell PM (2002). Mission Aligned Management and Allocation: A Successfully Implemented Model of Mission-based Budgeting. Academic Medicine.

[2] Mallon WT (2009). Introduction: The history and legacy of mission-based management. Academic Medicine, Management Series: Mission-Based Management.

[3] Nutter DO, Bond JS, Coller BS, D’Alessandri RM, Gewertz BL, Nora LM, Perkins JP, Shomaker S, Watson RT (2000). Measuring faculty effort and contributions in medical education. Acad Med.

[4] Holmes EW, Burks TF, Dzau V, Hindery MA, Jones RF, Kaye CI, Korn D, Limbird LE, Marchase RB, Perlmutter R, Sanfilippo F, Strom BL (2000). Measuring contributions to the research mission of medical schools. Acad Med.

[5] D’Alessandri RM, Albertsen P, Atkinson BF, Dickler RM, Jones RF, Kirch DG, Longnecker DE, McAnarney ER, Parisi VM, Selby SE, Stapczynski JS, Thompson JW, Wasserman AG, Zuza KL (2000). Measuring contributions to the clinical mission of medical schools and teaching hospitals. Acad Med.

[6] Mallon MT, Jones RF (2002). How do medical schools use measurement systems to track faculty activity and productivity in teaching?. Acad Med.

[7] Howell LP, Hogarth MA, Anders TF (2002). Creating a mission-based reporting system at an academic health center. Acad Med.

[8] Korn D (1996). Reengineering academic medical centers: reengineering academic values?. Acad Med.

[9] Watson RT, Romrell LJ (1999). Mission-based budgeting: removing a graveyard. Acad Med.

[10] Ruedy J, MacDonald NE, MacDougall B (2003). Ten-year experience with mission-based budgeting in the faculty of medicine of Dalhousie University. Acad Med.

[11] Howell LP, Hogarth MA, Anders TF (2003). Implementing a mission-based reporting System at an academic health center: a method for mission enhancement. Acad Med.

[12] Sloan TB, Kaye CI, Allen WR, Magness BE, Wartman SA (2005). Implementing a simpler approach to mission- based planning in a medical school. Acad Med.

